# Isolation and Characterization of Protein Fractions for Valorization of Sacha Inchi Oil Press-Cake

**DOI:** 10.3390/foods12122401

**Published:** 2023-06-17

**Authors:** Erwin Torres-Sánchez, Blanca Hernández-Ledesma, Luis-Felipe Gutiérrez

**Affiliations:** 1 Facultad de Ciencias Agrarias, Universidad Nacional de Colombia, Carrera 30 No. 45-03, Bogotá 111321, Colombia; egtorressa@unal.edu.co; 2 Instituto de Investigación en Ciencias de la Alimentación (CIAL, CSIC-UAM, CEI-UAM+CSIC), Nicolás Cabrera 9, 28049 Madrid, Spain; bhernandez@ifi.csic.es; 3 Instituto de Ciencia y Tecnología de Alimentos, Universidad Nacional de Colombia, Carrera 30 No. 45-03 Edificio 500A, Bogotá 111321, Colombia

**Keywords:** ATR-FTIR, biomass valorization, circular economy, techno-functional properties, *Plukenetia volubilis*, protein extraction, SDS-PAGE

## Abstract

The growing interest in plant-based food protein sources has provided opportunities for the valorization of agri-food by-products, driving the food industry towards more sustainable development. In this study, three extraction procedures (varying the pH value (7.0 and 11.0) and the addition of salt (0 and 5%)) were investigated to obtain seven different protein fractions (SIPF) from Sacha Inchi oil press-cake (SIPC), which were characterized in terms of their protein content, electrophoretic profile, secondary structure, and techno-functional properties. Extractions at pH 11.0 without salt addition produced the highest values of protein content, extraction yield, protein recovery, and protein concentration increase (84.0%, 24.7%, 36.5%, and 1.5-fold, respectively). Under these extraction conditions, the electrophoretic analysis indicated that most of the SIPC proteins were extracted. SIPF displayed an excellent oil absorption capacity (4.3–9.0 *w*/*w*), and interesting foam activity (36.4–133.3%). The solubility and emulsifying activity of the albumin fractions were significantly higher than those of the other fractions (~87 vs. <15.8%, and 280–370 vs. <140 m^2^/g, respectively). Correlation analysis showed that the secondary structure of the SIPF significantly influences their techno-functional properties. These results indicate that SIPC is a by-product of great potential for protein extraction processes, and as a valorization strategy for technical cycle solutions for the Sacha Inchi productive chain in the circular economy context.

## 1. Introduction

The increasing interest in plant-based ingredients, and the continuous demand for alternative food protein sources, are part of the recent consumer demands that have put pressure on food processors. Moreover, the threat of climate change and the need to ensure food security for the growing population are some of the driving forces moving the food industry towards the application of more sustainable processes, and the valorization of the agri-food by-products is becoming more relevant.

Sacha Inchi (SI) (*Plukenetia* spp.) is a plant of the *Euphorbiaceae* family with a great expansion in Central and South America and parts of South East Asia and South Africa [1,2]. SI kernels are rich in oil (35–60%), proteins (25–30%), essential amino acids, minerals, and vitamin E [3,4]. Because of their high oil content and richness in essential fatty acids (α-linolenic, ~50%; and linoleic, ~35%), SI kernels are mainly used for oil extraction [5,6]. The continuous industrial demand for polyunsaturated oils has promoted the growth of the SI oil industries, and in response to this, the main by-products of oil processing (the shell and the oil press-cake (SIPC)), which may represent up to 70% of the raw seeds [6], need to be valorized, in a circular economy context, since they are normally discarded or minimally used in animal feeding.

The protein content of the SIPC varies between 53 and 59% [7], which is higher than that recently reported in soybean meal varieties (42–50%) [8]. Moreover, SI proteins are considered of high biological value. They contain essential amino acids, including lysine, leucine, histidine, and phenylalanine [9]. However, SI proteins have scarcely been investigated. Albumins may represent 25% (*w*/*w*) of the SIPC, and their purity can exceed 90%. These predominant proteins have been extracted at neutral pH, and it has been reported that they are basic 3S proteins with a pI of approximately 9.4. They are composed of two glycosylated polypeptides (4.8% sugars) with estimated molecular weights (MW) of 32.8 and 34.8 kDa [10]. Furthermore, the extraction of proteins using the Osborne procedure resulted in the solubilization of over 90% of the SIPC proteins, which are represented by albumins (43.7%), globulins (27.3%), prolamins (3.0%), and glutelins (31.9%), with molecular weight ranges of 6–70 kDa [11]. Additionally, crude papain (pH 7.0 at 40 °C) and Calotropis proteases (pH 8.0 at 60 °C) have been employed for protein extraction from SIPC, recovering peptides with molecular weights ranging between <8 and 57 kDa [12]. In a recent study, the physicochemical, functional, and in vitro digestibility of SI protein isolates from Thailand and Peru were investigated, concluding that these isolates have potential applications as plant-based protein additives [9]. Taking into account that SI proteins could be used as ingredients in the food industry, and that protein demand is increasing [7], there is a need to investigate how the extraction procedures affect the properties of the protein fractions.

In this context, the aim of this study was to isolate and characterize seven protein fractions extracted from SIPC using three eco-friendly and cheap extraction procedures. The protein fractions were assessed for their protein content, their molecular weight using sodium dodecyl sulfate–polyacrylamide gel electrophoresis (SDS-PAGE), their secondary structure using Attenuated Total Reflectance Fourier Transform Infrared (ATR-FTIR) spectroscopy, and their oil absorption capacities (OAC), water absorption index (WAI), water solubility index (WSI), foaming and emulsifying properties, and color. Furthermore, a correlation analysis was performed to investigate the relationship between the secondary structure of these protein fractions and their functional properties. This new information could be useful for a better understanding of how SI proteins could be extracted and used in food formulations, and provide a technological route for the valorization of this important by-product of the Sacha Inchi productive chain, thus promoting the goals of sustainable development.

## 2. Materials and Methods

### 2.1. Materials

SIPC was generously provided by SumaSach’a (Mosquera, Cundinamarca, Colombia). BioLegend Inc. provided the protein ladder Prime-Step (San Diego, CA, USA). ITW Reagents PanReac-AppliChem (Darmstadt, Germany) supplied acrylamide, butanol, hydrochloric acid, mercaptoethanol, methanol, and tetramethylethylenediamine (TEMED). Loba-Chemie (Tarapur, India) provided bis-acrylamide, bromophenol blue, Coomassie Brilliant Blue R-250, glacial acetic acid, glycerin, glycine, persulfate ammonium (APS), SDS, and sodium hydroxide. Merck KGaA (Darmstadt, Germany) supplied the tris-hydroxymethyl aminomethane (TRIS).

### 2.2. SIPC Adequacy

Two batches (2.5 kg each) of SIPC were ground using an impact pulverizer Raymond Screen Mill (Combustion Engineering Inc., Raymond Division, Windsor, CT, USA), and then, mechanically sieved using an American Society for Testing and Materials (ASTM) sieve system. Particles with a size of 250 µm were retained for the study. The obtained flour was defatted (SIPCD) under continuous stirring (1000 rpm) at room temperature (RT) (~20 °C) for 2 h, using petroleum benzine in a 1:10 ratio (*w*/*v*). The slurry was vacuum filtered on a Whatman No. 4 paper, and left to dry overnight at RT. The dried SIPCD was vacuum packaged and stored at −20 °C until further analysis.

### 2.3. Proximal Composition

The proximal composition of both SIPC and SIPCD was determined according to official procedures [13]: moisture (Method 934.01); the crude protein (Method 978.04) was calculated from total nitrogen using the conversion factor of 5.71 (Sathe et al., 2012); the ash and fat contents (Methods 930.05 and 930.09, respectively); the total dietary fiber was determined following the gravimetric-enzymatic method (991.43) using a kit (TDF-C10, Sigma Aldrich^®^, St. Louis, MO, USA), and carbohydrates were determined by difference.

### 2.4. Isolation of the Protein Fractions

**Figure 1:**
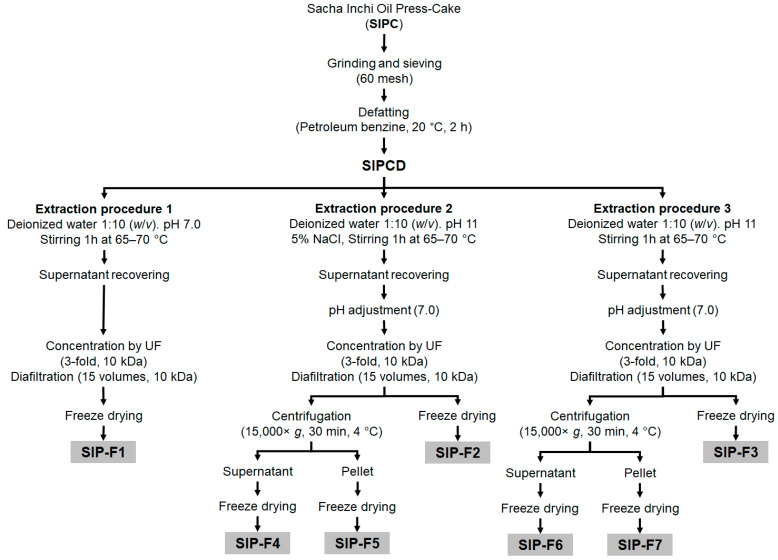
Sacha Inchi protein fractions’ extraction procedures.

Figure 1 presents the extraction procedures evaluated for the isolation of the protein fractions (SIP-F1 to SIP-F7) from the SIPCD.

In all extraction procedures, the SIPCD was dispersed in deionized water in a 1:10 (*w*/*v*) ratio, under continuous stirring for 1 h at 800 rpm at temperatures between 65 and 70 °C. Neutral (pH 7.0, extraction procedure 1) and alkaline conditions (pH 11.0) with (extraction procedure 2) or without (extraction procedure 3) salt addition (5% NaCl) were investigated. After extraction, the supernatants were recovered by filtration with cheesecloths, neutralized with NaOH 1 M at pH 7.0 (extraction procedures 2 and 3), and subsequently concentrated and diafiltrated with deionized water (0.3 µS/cm) on a 10 kDa molecular weight cut off Hydrosart ultrafiltration membranes (Sartorius^®^, Vivaflow 200-VF20H0, Göttingen, Germany). The concentrated and diafiltrated supernatants were freeze dried and the SIP-F1, SIP-F2, and SIP-F3 were obtained. In the case of the concentrated and diafiltrated supernatants from the extraction procedures 2 and 3, a centrifugation step (15,000× *g*, 30 min, 4 °C) was also applied to separate the true albumins (SIP-F4 and SIP-F6) from true globulins (SIP-F5 and SIP-F7). Diffusion in a macromolecule solution leads to uniform distribution. Centrifugation separates proteins based on their sedimentation rates, with larger particles forming a pellet and smaller particles remaining in the supernatant, as previously reported [11].

The extraction conditions were chosen for their effects on protein solubility and extraction efficiency [9,14,15]. The pH 7 was chosen as a neutral condition to extract soluble proteins at a physiological pH, and it was previously reported for Inca Peanut Albumin extraction [7,10]. On the other hand, pH 11 may enhance the solubility of major proteins in SIPC. A 5% salt concentration was chosen to create conditions that favor the selective solubilization of reported globulin proteins [16]. The temperature range of 65–70 °C falls within the typical extraction range for proteins without significant denaturation or degradation [17]. All the extraction procedures were carried out in duplicate. The obtained freeze-dried SIP-F1 to F7 were weighed and stored at −20 °C until further use.

The protein content of the protein fractions was determined by the nitrogen combustion method (N × 5.71) using an elemental analyzer (LECO 630-100-100 TruSpec CN analyzer, St. Joseph, MI, USA). The extraction yields, protein recovery, and the protein concentration increase obtained for each of the extraction procedures were calculated using Equations (1)–(3), respectively:

Extraction yield (%) = [Lyophilized protein fraction (g)/Initial SIPCD (g)] × 100,(1)

Protein recovery (%) = [(Protein content in the protein fraction (%) × lyophilized mass (g))/(Protein content SIPCD (%) × initial SIPCD mass (g)] × 100,(2)

Protein concentration increase (fold) = [Protein concentration in protein fraction %]/[Protein concentration in SIPCD %],(3)

### 2.5. Characterization of the Protein Fractions

#### 2.5.1. Sodium Dodecyl Sulfate–Polyacrylamide Gel E (SDS-PAGE)

The protein profiles of the SIP-F1 to F7 were analyzed by SDS-PAGE. Samples were prepared by adding a buffer, composed of 0.05 M Tris-HCl (pH 6.8), SDS (1.6%, *w*/*v*), bromophenol blue (0.002%, *w*/*v*), β-mercaptoethanol (2%, *v*/*v*), and glycerol (8%, *v*/*v*), incubated at 95 °C for 5 min, and centrifuged (3000× *g*, 5 min, 4 °C). Electrophoretic analyses were carried out in a Mini-V Vertical Electrophoresis Apparatus (Apogee Electrophoresis, Baltimore, MD, USA), using polyacrylamide 4 and 12% Bis-Tris hand cast gels. A total of 10 µL of sample (~75 µg of protein) was loaded per lane. Tris-Glycine 1× buffer was used for separation, and electrophoretic migration was carried out at a voltage of 100 V for 5 min, and then 130 V for 90 min. Gel staining was performed with Coomassie blue (R-250). The Prestained Broad Range Protein Ladder (BioLegend, San Diego, CA, USA) was used as marker. The gel image was taken using the Gel Doc XR+ system gel reader (Bio-Rad Laboratories, Hercules, CA, USA) and processed with Image Lab™ (Version 6.0.1 build 34, Standard Edition, Bio-Rad Laboratories, Inc., 2017).

#### 2.5.2. Protein Secondary Structure Analysis

ATR-FTIR analyses of lyophilized SIPF were carried out on an FT/IR-4700 type A spectrometer (JASCO, Tokyo, Japan) with an accessory ATR Pro One with a single-reflection diamond crystal at a 45° incidence angle, and a triglycine sulfate (TGS) detector. A flat tip was used to obtain an intimate contact between sample and crystal, without pressure control. Spectra were recorded from 4000 to 400 cm^−1^ with a resolution better than 4 cm^−1^ at a scanning speed of 2 mm s^−1^. Measurements were carried out at RT, and the frequency values of each absorption band were obtained automatically by the software. The spectra were analyzed using OriginPro 2023 (OriginLab Corporation, Northampton, MA, USA). Deconvolution and secondary derivative were applied to the range of 1700–1600 cm^−1^, which was attributed to the amide I band in protein FTIR spectrum [18]. The FTIR spectra were processed by using data normalization from 0 to 1. Baseline correction was performed using the Shirley baseline mode and subtracting the baseline [19,20]. Peak analyzer (using the Levenberg–Marquardt algorithm) was used to perform nonlinear fitting of the peaks in the spectral data. Positive hidden peaks were detected using a second derivative method followed by smoothing with the 7–9 points of window Savitzky–Golay function with polynomial order of 2 [21,22]. Finally, the corresponding peaks were adjusted, and the area measured with the Gaussian function: intermolecular β-sheet (1627–1610 cm^−1^), intramolecular β-sheet (1642–1628 cm^−1^), random coil (1650–1643 cm^−1^), α-helix (1659–1650 cm^−1^), and β-turn (1700–1660 cm^−1^). The areas of all the component bands assigned to a given conformation were added up and divided by the total area [21].

#### 2.5.3. Oil Absorption Capacity (OAC)

OAC was determined following the method described by Zheng et al. [23]. The lyophilized SIP-F1 to SIP-F7 samples were weighed (100 mg, *M*_0_) into pre-weighed 2 mL centrifuge tubes and thoroughly mixed with 1 mL (*M*_1_) of commercial sunflower oil using a vortex. The samples were allowed to stand for 30 min. The protein–oil mixture was centrifuged (4000× *g*, 20 min, 4 °C), the supernatant was discarded, and the weight of the precipitates was recorded (*M*_2_). OAC was computed utilizing Equation (4):

(4) $$\mathrm{OAC}\;(w/w)=\left[\frac{M_{1}-M_{2}}{M_{0}}\right],$$

#### 2.5.4. Water Absorption Index (WAI) and Water Solubility Index (WSI)

WAI and WSI were determined according to the method proposed by Jiapong and Ruttarattanamongkol [24]. Sample (100 mg) was weighed into pre-weighed centrifuge tube, dispersed in 1 mL of distilled water, shaken by vortex, and left to hydrate for 30 min before centrifugation (4000× *g*, 20 min, 4 °C). The supernatant was decanted into a pre-weighed Petri dish, and dried at 105 °C. The WAI and WSI were calculated employing Equations (5) and (6), respectively:

WAI (*w*/*w*) = [*Weight of sediment*/*Weight of sample*], (5)

WSI (%) = [*Weight of dried solids from the supernatant*/*Weight of sample*] × 100, (6)

#### 2.5.5. Foaming Properties

Foaming properties were determined according to the reported protocol by Haque and Kito [25]. A total of 1 g of each sample was whipped with 34 mL of distilled water in an Ultra Turrax^®^ T18 basic (IKA^®^, Staufen, Germany) at 10,000 rpm for 1 min, and the mixture was poured into a 100 mL graduated cylinder. Foam activity (FA) and foam stability (FS) were determined using Equations (7) and (8), respectively:

(7) $$\mathrm{FA}\;(\%)=\left[\frac{L_{1}+F_{1}}{L_{0}}-1\right]\times 100,$$

(8) $$\mathrm{FS}\;(\%)=\left[\frac{100\times F_{2}}{F_{1}}\right],$$

where *L*_0_ and *L*_1_ are the volumes of the mixture before and after shaking, respectively. *F*_1_ and *F*_2_ are the volumes of the foams after shaking and after standing for 30 min, respectively. Considering that different proteins may produce foams with different gaseous content, the foam density (FD) was also considered, and it was calculated using Equation (9):

(9) $$\mathrm{FD}=\left[\frac{100\times (L_{0}-L_{1})}{F_{1}}\right],$$

#### 2.5.6. Emulsifying Properties

The emulsifying activity index (EAI) and emulsion stability index (ESI) were determined by the turbidimetric technique described by Khuwijitjaru et al. [26], at three different amounts of each sample: 50, 100, and 200 mg. For the emulsion formation, 6 mL of 0.2% sample dispersion in 0.05 M Tris-HCl buffer (pH 7.5) and 2 mL of commercial sunflower oil were homogenized in an Ultra Turrax for 1 min at 15,000 rpm. An aliquot (50 µL) was taken after 0 and 10 min, diluted (1:100, *v*/*v*) in 0.1% (*w*/*v*) SDS solution, and after mixing by vortex, the absorbance was read at 500 nm using a UV-vis spectrophotometer (GENESYS 20, Thermo Fisher Scientific, Waltham, MA, USA). The turbidity (*T*), EAI, and ESI (after 10 min) of emulsions were determined using Equations (10)–(12), respectively:

(10) $$T=\left[\frac{2.203\times A}{b}\right],$$

(11) $$\mathrm{EAI}\;(m^{2}/g)=\left[\frac{2\times T\times DF}{\theta \times C\times 10,000}\right],$$

(12) $$\mathrm{ESI}\;(\min)=\left[\frac{T\times \Delta t}{\Delta T}\right],$$

where *A* is the absorbance at 500 nm, *b* is the path length of the cuvette (0.01 m), *DF* is the dilution factor (100), *θ* is the volumetric fraction of oil (0.25), *C* is the weight of protein fraction per unit volume (0.157 g/mL), 10,000 is the correction factor for square meters, Δ*T* is the change in turbidity (value at 0 and after 10 min), and Δ*t* is the time interval (0.17 h).

#### 2.5.7. Color

The instrumental color of the protein fractions was determined using a bench-top spectrophotometer colorimeter CM-5 with 10° viewing angle, D65 illuminator, and 30 mm aperture diameter (Konica Minolta, Osaka, Japan). The CIE-Lab (L*, a*, b*) parameters were determined [6], as the average of two measurements per sample. The total color difference (ΔE) was determined using Equation (13):

(13) $$\Delta E=\sqrt{{\Delta L}^{*2}+{\Delta a}^{*2}+{\Delta b}^{*2}},$$

### 2.6. Experimental Design and Statistical Analysis

The experiments were carried out under a completely randomized design. Each parameter was evaluated independently. All the measurements were performed at least in duplicate. The analysis of variance (ANOVA) and test of significance (least significant differences test, LSD) were performed using the SAS software version 9.4 (SAS Institute Inc., Cary, NC, USA). The homogeneity of variances was tested using the Levene test. The normality of the residuals was tested using a Shapiro–Wilks test. Differences were considered statistically different at *p* < 0.05.

## 3. Results and Discussion

### 3.1. Proximal Composition

The contents of moisture, protein, fat, total dietary fiber, ash, and carbohydrates of the SIPC and SIPCD are presented in Table 1. As noticed, the press-cakes of Sacha Inchi can be considered important sources of proteins (54.5–56.9%), dietary fiber (13.9–18.2%), and minerals (6.1–6.4%). Defatting with petroleum benzine reduced the lipid content of the SIPC from 6.8 to 1.7%. Carbohydrates and moisture ranged between 6.6 and 9.7% and around 9.0 to 10.0%, respectively. In general, these compositional data are between the normal ranges reported for SI [7,27,28,29], and similar to those of soybean meals from different countries [8], with the latter being the most popular protein sources for application in both the livestock and human food industries.

### 3.2. Isolation of the Protein Fractions

In this study, we investigated three different protein extraction procedures (Figure 1) for obtaining and characterizing seven protein fractions (SIP-F1 to SIP-F7) from SIPCD. The protein content, extraction yield, protein recovery, and protein concentration increase of these fractions are presented in Table 2.

The protein content of the isolated fractions ranged between 60.5 and 93.1%, while the extraction yields and the protein recovery varied from 4.9 to 24.7%, and from 5.2 to 36.5%, respectively. The concentration of proteins in the obtained fractions increased from 1.1 to 1.6. Raising the pH value of the extraction medium from 7.0 to 11.0 (SIP-F1 vs. SIP-F2 and SIP-F3) enhances all the response variables, in agreement with previous studies that reported that extractions under alkaline conditions promoted the extraction yield and protein isolate purity [30]. Although the addition of NaCl allowed the extraction yield to be doubled (SIP-F1 vs. SIP-F2) for samples extracted at pH 7.0, the isolation of proteins from the SIPCD displayed the highest yield (36.5%) at a pH value of 11.0 without salt addition (SIP-F3). Under these extraction conditions, the isolation of the globulin fraction (SIP-F7) yielded 33.1%. The highest protein content (93.1%) was obtained in the globulin fraction SIP-F5, possibly because the addition of salt contributes to the separation of globulins from albumins through centrifugation, as reported previously [10]. However, the yield of its extraction procedure was quite low (7.0%). The albumin fractions (SIP-F4 and SIP-F6) displayed similar protein contents (~74%), but their extraction procedures showed the lowest extraction yields (3.0 and 2.2%, respectively).

The protein contents found in this study for SIPF are in agreement with the previous findings of Suwanangul et al. [15], who reported 89.4% protein content in which a Sacha Inchi protein isolates. The variation of the data could be explained because the solubility of proteins is affected by many factors such as structure, concentration, and other parameters, such as pH, temperature, and the type and content of salts [31]. The superior performance (extraction yield, protein content, protein recovery, and protein concentration increase) of SIP-F3 could be due to the high levels (~11%) of sulfur-containing amino acids (cysteine ~9% + methionine ~1.5%) present in the albumin proteins [16]. The disulfide bonds and sulfhydryl groups play an important role in the aggregation of proteins, and the high performance could be a consequence of the presence of protein aggregates formed by hydrophobic and thiol–disulfide interactions [31].

Additionally, alkali conditions and a high temperature (60–70 °C) may cause protein denaturation and an increase in the surface hydrophobicity, decreasing the water–protein interactions and favoring the extraction process [32]. The protein content, intermediate extraction yield, and protein recovery obtained in the SIP-F2 treatment, could be due to the combined role of the NaCl and NaOH in the extraction media. While NaCl modifies the ionic strength of the media, NaOH alters the protein structure and the interactions, which could have contributed to the protein recovery, extraction yield, and protein content of this fraction [32].

On the other hand, the low protein content and performance of the SIP-F1 treatment was possibly due to the absence of ions (NaCl or NaOH) that competed with the protein by water molecules in order to increase the protein–protein interactions, which allows the protein to unfold and the formation of insoluble aggregates, and finally their precipitation [31].

### 3.3. Characteristics of the Protein Fractions

#### 3.3.1. Electrophoretic Profiles

**Figure 2:**
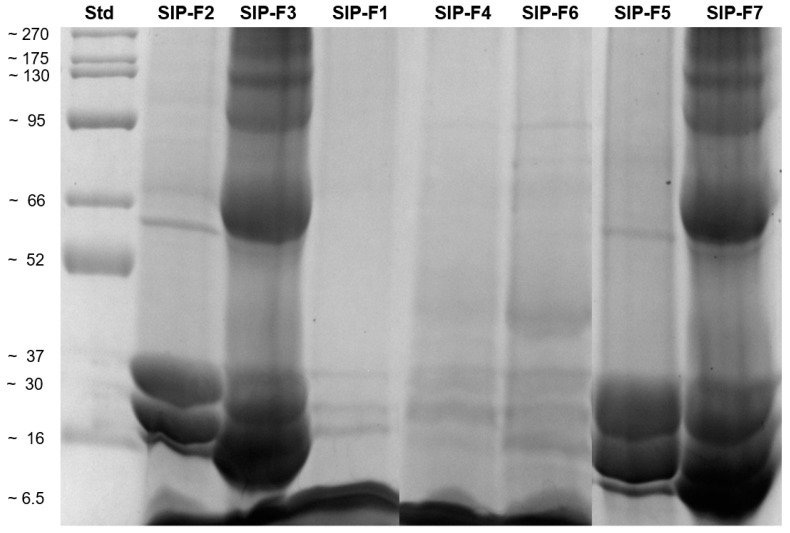
SDS-PAGE analysis of Sacha Inchi protein fractions. (Std): protein marker standard; SIP-F1 (0% NaCl; pH: 7.0; T 65–70 °C; 1 h); (SIP-F2): (5% NaCl; pH: 11; T 65–70 °C; 1 h); SIP-F3 (0% NaCl; pH: 11.0; T 65–70 °C; 1 h); SIP-F4 and SIP-F5: true albumins and true globulins of SIP-F2, respectively; SIP-F6 and SIP-F7: true albumins and true globulins of SIP-F3, respectively.

Figure 2 shows the band profiles of the seven SIPF obtained. The same profile of polypeptides with molecular weights of 59, 37, and 30 kDa was observed in SIP-F2 and SIP-F5. One dimensional analysis of these fractions (judged qualitatively based on band width and intensity) showed that the major polypeptides (16–37 kDa range) corresponded to the albumin fraction, and to a lesser extent to globulins (59–88 kDa range), as previously reported [10,11].

Similarly, SIP-F3 and SIP-F7 showed the highest number of polypeptides, and the same profile with molecular weights of 175, 130, 95, 88, 66, 59, 37, 30 and 28 kDa. The analysis of these fractions allowed it to be deduced that the use of alkaline water (pH 11.0) and high temperatures (60–70 °C) was quite efficient for the extraction of most of the SI proteins such as globulins, albumins, prolamins, and glutelins, as previously reported in proteins obtained from SI seeds using the Osborne extraction method [11,16]. Under this extraction method, proteins with high molecular weights (175, 130, 95, and 88 kDa) that had not previously been reported in the literature were evidenced. These protein bands were possibly globulins/storage proteins, similar to those found in pulse seeds. For example, vicilins are identical among pulses, but their molecular weights vary from 133 to 140 kDa in cowpea, 136 to 150 kDa in kidney beans, 162 kDa in mung bean, 173 kDa in red bean, 155 kDa in pea, and 163 kDa in fava/faba beans [33]. The expression of these proteins in the electrophoretic profile could be due to the geographical origin of the SI kernels. Thus, these results are consistent with a study that compared SI seeds from different locations in Peru, reporting protein electrophoretic profiles that were not expressed in all ecotypes and regions, despite being from the same species *Plukenetia volubilis* [34].

Likewise, extractions with deionized water at pH 7.0, allowed three polypeptides with molecular weights of 29, 30, and 32 kDa to be obtained, in a weak but purified form that corresponds to the albumin protein fraction previously reported [10]. In the case of the protein fractions SIP-F4 and SIP-F6, a similar profile was evident, with polypeptides with molecular weights of 95, 82, 70, 66, 45, 32, 30, 28, and 16 kDa. In these protein fractions, a 45 kDa band was visible, while it did not appear in the rest of the fractions. It probably corresponded to 11S globulin, while the 70 kDa band corresponded to 7S globulin, previously reported in SI kernels [35].

#### 3.3.2. Fourier Transform Infrared (FTIR) Spectroscopy

**Figure 3:**
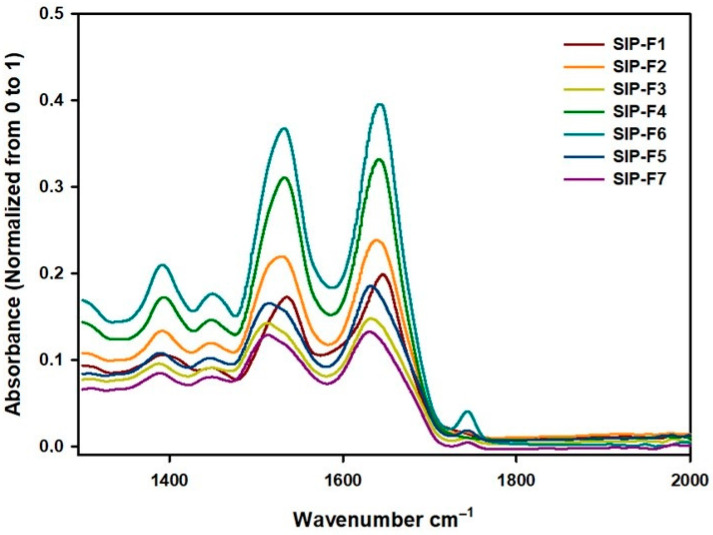
FTIR spectra for Sacha Inchi protein fractions. SIP-F1 (0% NaCl; pH: 7.0; T 65–70 °C; 1 h); SIP-F2 (5% NaCl; pH: 11.0; T 65–70 °C; 1 h); SIP-F3 (0% NaCl; pH: 11.0; T 65–70 °C; 1 h); SIP-F4 and SIP-F5: true albumins and true globulins of SIP-F2, respectively; SIP-F6 and SIP-F7: true albumins and true globulins of SIP-F3, respectively.

Figure 3 shows the region of interest in the study of the protein secondary structure, between 2000 and 1300 cm^−1^. The amide I band, which has the strongest absorption of infrared light, was found at ~1650 cm^−1^. It is primarily caused by stretching vibrations of C=O coupled weakly with C–N stretch and N–H bending. It is the most sensitive to structural changes and is the most used in secondary structure protein analysis. The amide II band occurs at ~1550 cm^−1^ and is mainly derived from the C–N stretch along with N–H in-plane bending. Lastly, the amide III band is found at ~1300 cm^−1^; the vibrations responsible for this band are a complex mix of N–H bending and C–N stretching along with deformation vibrations of C–H and N–H. The bending of the –OH groups at ~1420 cm^−1^ and ~1380 cm^−1^ correspond to the COOH functions of carboxylic acids and alcohols, respectively. A straight baseline between 2000 and 1750 cm^−1^ is a criterion for determining whether the absorption by water was correctly subtracted [18,22].

As can be seen in Figure 3, the FTIR spectroscopy for SIPF produced spectra with similar patterns at amide I, II, III, and –OH groups regions, with some differences, attributed to the protein fraction and extraction method, in peak intensities due to the repetition of the same functional groups, which leads to relatively larger and more intense peaks [36].

The underlying individual components of the secondary structure could not be seen in the amide I band. This was because the width of the bands of different components was greater than the separation between the peaks of individual components’ bands [21]. Therefore, the second derivative of the spectrum was used to identify the hidden peaks. By performing the fit on the spectra, the overlapping hidden peaks were identified and are reconstructed in Table 3.

SIP-F1 exhibited α-helix structures in a proportion of ~45%, intramolecular β-sheet of ~29%, β-turn of ~23%, and intermolecular β-sheet of ~4%, and did not exhibit random coil structures. This result was consistent with the reported proportions of α-helix, intramolecular β-sheet, β-turn, and intermolecular β-sheet (28%, 20%, 18%, and 9%, respectively), except for the random coil structures (25%), for a fraction of SI albumins reported by [16].

For SIP-F2, an alkaline pH in combination with NaCl addition caused the vanishing of the intramolecular β-sheet and α-helix structures, and significantly increased the random coil structures (~58%) and intermolecular β-sheet (~20%). This result was in accordance with the findings reported by [37], who studied the effect of basic pH and chaotropic salts’ addition on the secondary structures of *Dolichos lablab* and *Phaseolus calcaratus* vicilins. The study concluded that alkaline conditions (pH 11.0) decreased the content of the α-helix and β-sheet structures, while the presence of chaotropic salts (1 M NaCl) increased the content of the random coil structures.

In the present study, the use of NaCl (5.0%) reduced the stability of helix structures and affected the stability of proteins by destroying the electrostatic interactions between charged amino acid residues [38], causing an intramolecular reorganization. Otherwise, the use of an alkaline pH without the addition of NaCl in the SIP-F3 caused the disappearance of the intermolecular β-sheet and α-helix structures, and significantly increased the intramolecular β-sheet structures (~67%). In this regard, the alkaline pH facilitated the conversion of sulfhydryl groups into intermolecular and intramolecular disulfide bonds [39].

In addition, the albumin fractions (SIP-F4 and SIP-F6) exhibited a significantly high proportion of random coil structures (~58% and 71%, respectively), and low proportions of β-turn structures (~15% and 17%, respectively). In these two fractions, there were no differences in the intermolecular β-sheet structure (~11%), and SIP-F4 showed intramolecular β-sheet and α-helix structures (11% and 5%, respectively) that were not present in SIP-F6. This implied that these albumin fractions had fewer nonrepetitive regions in the polypeptide backbone conformation [40].

On the other hand, the globulin fractions SIP-5 and SIP-F7 exhibited a high content of intermolecular β-sheet, intramolecular β-sheet, and β-turn structures (ranging from 11–17%, 32–39%, and 24–28%, respectively), and an intermediate content of α-helix structures (19–27%). These protein fractions displayed a secondary structure similar to those reported by Mao et al. [41] for walnut protein isolates and concentrates (34.9% α-helix, 11% β-sheet, 23.3% β-turn, and 32% random coil), for rice globulins (30% α-helix, 50% β-sheet, and 20% random coil), and soybean 7S and 11S globulins (14.5 and 17% for α-helix, 45.6 and 47.3% for β-sheet, and 23.8 and 19.3% for β-turn, respectively) [33].

### 3.4. Functional Properties of the SIPF

The functional properties of proteins define their application in food matrices. Oil and water absorption, solubility, emulsifying properties, and foam formation are considered the most important functional properties of protein isolates in food industries [33]. The techno-functional properties of the SIPF are presented in Table 4.

#### 3.4.1. OAC, WAI, and WSI

The OAC is related to the binding of fat by nonpolar side chains of proteins, and depends on the amino acid profile of proteins, especially the fraction of hydrophobic residues that interact with the hydrocarbon chains of lipid molecules (Sathe et al., 1982). This ability of proteins is very important for the formulation of products that demand a high OAC since it improves flavor retention, smoothness, and mouthfeel [42]. Likewise, it decreases the development of oxidative rancidity and consequently increases stability during storage [43]. A protein isolate has an excellent OAC if it reaches a value of ~300%.

In this sense, all SIPF had an excellent OAC with values that ranged from 4.3 to 9.0 (*w*/*w*, g of oil per gram of freeze-dried sample) (Table 4). These results were higher than those reported by Mercado et al., 2015 [44], in which a protein isolate from SI showed an OAC of 2.7 (*w*/*w*), or equivalently, 271%. It was also superior to the OAC reported for protein concentrates from oilseeds such as soybean, *Trichilia emetica*, *Trichilia dregeana*, and *Theobroma grandiflorum* Schum (466%, 200%, 250%, and 577%, respectively) [36,45]. The protein fractions F3 and F6-F7, extracted under alkaline conditions and through centrifugation, respectively, exhibited the highest OACs. The extraction process using an alkaline pH and subsequent concentration of globular proteins through centrifugation may be responsible for the OAC of these protein fractions. The denaturation process that occurs in an alkaline environment caused globular proteins to unfold, facilitating the binding of hydrophobic amino acids to fat [46].

Otherwise, WAI may be defined as the ability of proteins to physically hold water against gravity, through bound water, hydrodynamic water, capillary water, and physically entrapped water, which usually depends on the availability of hydrophilic groups (−OH, −NH_2_, −COOH, and –SH) [24]. Protein with good WAI (~5 *w*/*w*) can be applied in the prevention of water loss in bread and cakes and to increase yields of cured sausages, canned food, and frozen products [47]. The WAI of the SIPF ranged from 0.5 to 5.0 (*w*/*w*, g of water per gram of freeze-dried sample) (Table 4), with the SIP-F2, SIP-F3, SIP-F5, and SIP-F7 treatments having values between 3 and 5.0 *w*/*w*. These results are consistent with the WAI reported for samples obtained from different varieties of pulse plants, such as kidney bean, field pea, and cowpea (1.6–4.8, *w*/*w*), *Vicia faba* protein isolate (2.6, *w*/*w*), chickpea protein isolates (2.3–3.5, *w*/*w*), and lentil protein isolates (2.8–2.9, *w*/*w*) [33].

On the other hand, the WSI primarily depends on the balance between hydrophilic and hydrophobic residues in the protein structure. For consumers, the rapid and complete reconstitution of powdered food is one of the main quality indicators for ready-to-eat products [48]. The WSI values of the SIPF ranged from 2% to 82% (Table 4). The albumin fractions SIP-F4 and SIP-F6 exhibited significantly higher values (~86.5%) compared to the others’ SIPF. Conversely, these same protein fractions possess the lowest WAI values. This can be explained by the fact that a high protein solubility does not necessarily imply a high WAI value [41]. Additionally, it has been demonstrated that SI albumins are glycosylated polypeptides [10], and these proteins likely have abundant hydrophilic groups (such as polar or charged side chains) that promote their solubility. The lowest solubility values were observed in the globulin SIP-F5 and SIP-F7 fractions. One potential explanation for the low WSI of these fractions could be related to the centrifugation, diafiltration, and freeze-drying steps performed, which could lead to the formation of large aggregates [49]. Additionally, the presence of a high concentration of hydrophobic amino acids (~34%) [16] in the structure of these fractions could explain their higher tendency towards oil absorption and lower affinity for water, while the low levels of total hydrophobic amino acids in the globulin fractions SIP-F4 and SIP-F6 (~32%) [16] could explain their higher solubility and lower affinity for OAC and WAI.

#### 3.4.2. FA, FS, and FD

Foams are defined as thermodynamically unstable biphasic colloidal systems, where the gas phase is dispersed into a continuous liquid phase. The FA is related to the ability of proteins to decrease the surface tension, while FS depends on the extension of protein–protein interactions occurring in the intermolecular network, and FD is the gaseous content in different foams [25,42]. Foaming properties play a crucial role in determining the applications of proteins in food products where aeration and high-volume expansion are required. These properties are particularly important in products such as beer, cakes, fudges, confectionery items, whipped toppings, soufflés, mousses, ice cream mixes, and more [33].

Table 4 shows the FA, FS, and FD of the different SIPIF. The SIP-F1, SIP-F2, and SIP-F3 treatments created foams with a significantly high FA, ranging from 88% to 133%, and ~122% for SIP-F7, while the SIP-F4, SIP-F6, and SIP-F5 treatments had foam overruns ranging from 36% to 62%, showing a significantly better foam ability for the SIPF in which albumins were not separated from globulins by centrifugation. The SIP-F1, SIP-F2, and SIP-F3 treatments were also better at creating smaller air bubbles, with low FD values. However, the FS (evaluated at 30 min) was similar for most treatments (~88%), except for SIP-F2 and SIP-F3, where only the foam level was reduced by approximately 64%. The lower FA of globulins is attributed to their reduced ability to unfold or reorient at the air–water interface, limiting their capacity to effectively encapsulate air bubbles.

On the other hand, albumins exhibited a higher FA due to improved protein unfolding, which enabled better stabilization of air bubbles and promoted foam formation [33]. The results of the present study are consistent with those reported for protein fractions (albumins and globulins) obtained from mung bean, Bambara groundnut, and yellow pea, where the water-soluble protein fractions, specifically the albumins, showed a higher FA than the globulin fractions (257% to 281% vs. 12% to 61%). Additionally, the albumin fractions were more effective at creating smaller air bubbles compared to the globulin fractions (0.06 mm vs. 0.20 mm, respectively). Furthermore, the albumin fractions showed exceptionally higher FS than globulins (foam half-life times between 240 and 314 min vs. 70 min) [49]. In pulse proteins, higher FA and FS values have been reported for albumins compared to globulins. For example, albumins derived from lentils showed high FA values of 76.7% and FS values of 66.7%, while globulins exhibited lower FA values of 16.7% and FS values of 6.7%. Similarly, albumins from horse gram demonstrated FA values of 79% and FS values of 55%, whereas globulins had FA values of 8.9% and FS values of 4.4%. These findings indicate that albumins had superior foam-forming properties compared to their corresponding globulin fractions [33].

#### 3.4.3. EAI and ESI

An emulsion may be defined as a dispersion of two immiscible liquids in which one liquid is dispersed in the form of globules within the continuous phase of another liquid. Such a system is thermodynamically unstable because of increased interfacial surface tension. The ability of proteins to stabilize an emulsion is regarded as one of the most important functional properties that decide their applications in food products such as comminuted meats, doughs, coffee/tea whiteners, ice creams, cakes, or mayonnaise [33]. The ability of a protein to form an emulsion can be described by the EAI as an appraisal of the interfacial area stabilized per unit weight of the protein, and the stability of the emulsion over a specific time is evaluated by the ESI.

**Figure 4:**
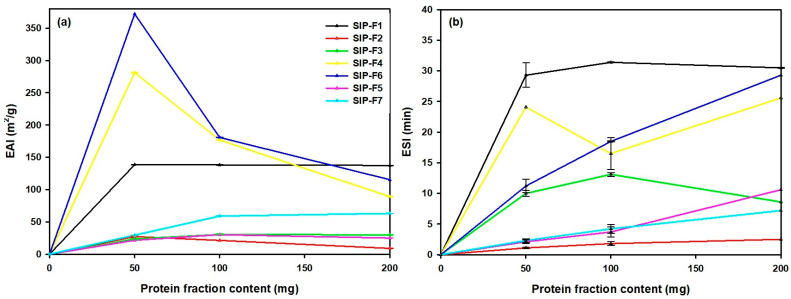
Emulsifying properties of Sacha Inchi protein fractions (SIPF). (**a**) Emulsifying activity index (EAI) and (**b**) emulsifying stability index (ESI) at 50, 100, and 200 mg. SIP-F1 (0% NaCl; pH: 7.0; T 65–70 °C; 1 h); SIP-F2 (5% NaCl; pH: 11.0; T 65–70 °C; 1 h); SIP-F3 (0% NaCl; pH: 11.0; T 65–70 °C; 1 h); SIP-F4 and SIP-F5: true albumins and true globulins of SIP-F2, respectively; SIP-F6 and SIP-F7: true albumins and true globulins of SIP-F3, respectively. The symbol conventions in (**a**,**b**) are identical.

Figure 4 shows the emulsification properties in SIPF. In general, emulsification was largely influenced by protein fraction and the amount evaluated.

For example, for 50 mg, the EAI of albumin protein fractions (SIP-F4 and SIP-F6) were significantly higher compared to the other fractions (Figure 4a). The better EAI of albumin fractions might be due to its quicker adsorption rate at the oil–water interface and more flexible structure than others [50]. However, a decrease in the EAI from 370 to 90 m^2^/g of albumin fractions at 100 and 200 mg was recorded. At higher SIPF concentrations, less emulsion activity was obtained, possibly due to a higher protein content. As the protein concentration increased, the time required for the molecules to form a stable film at the oil–water interface was generally reduced. However, by increasing the percentage of oil in the emulsion system, there was a greater possibility of creating new interfaces [26,51]. For SIP-F5 and SIP-F7, and SIP-F2 and SIP-F3 in different concentrations, EAI values between ~10 and ~60 m^2^/g were low due to the low solubility exhibited compared to albumin fractions. These results are consistent with the study conducted by [50] where the protein fractions of green pea and chickpea were evaluated and the values of the EAI for globulin and vicilin fractions were ~10 and ~55 m^2^/g, respectively.

For the ESI parameter, maximum values were obtained for SIP-F4, SIP-F6, and SIP-F1, (~25, ~29, and ~31 min, respectively) (Figure 4b). A similar trend was reported by Dias et al. (2022), where maximum (35 min) and minimum (18 min) ESI values were observed at pH 5.0 and 11.0, respectively, for protein extracts from full-fat almond flour, and the findings reported for the ESI values (~30 min) for water-soluble proteins obtained from soybeans [26] were slightly different from the ESI values reported for protein isolates from lentils, horse gram, kidney beans, field peas, and cowpeas, which ranged between 7.18 and 95.40 min [33]. Presumably, the protein fractions (SIP-F1 and SIP-F4) had a greater capability to anchor to the oil–water interface. This could be attributed to reduced repulsive forces between the protein molecules, resulting in a shorter distance between them. This favorable arrangement promoted protein adsorption and enhanced the viscoelasticity at the oil–water interface, thus increasing emulsion stability [47].

#### 3.4.4. Color

The CIE-Lab (L*, a*, b*) color parameters using rectangular coordinates for samples are shown in Table 5.

The values of lightness (L*) for SIPCD, SIP-F1, SIP-F2, SIP-F4, and SIP-F6 did not present significant differences. The red/green coordinate (a*) was significantly different among the samples; SIP-F5 and SIP-F7 differed from the others, exhibiting values with a tendency towards the red coordinate; a tendency towards the green coordinate was observed in SIPCD, SIP-F1, SIP-F2, SIP-F3, SIP-F4, and SIP-F6. Moreover, in the yellow/blue coordinate (b*), there were significant differences, with the albumin having the lowest value. The total color difference (ΔE) between the flours was not significantly different for most samples. The SIP-F7 sample had the greatest significant color difference, being generally darker, redder, and bluer in color than SIPCD. There are no reports in the literature of the color in SIPCD or its derived proteins flours. However, in a recent study, the color values (L* and b*) of the samples decreased, while the a* value increased when wheat flour was substituted for SIPC content at an increasing level [52]. Another study reported that when the amount of SIPC increased, the yellowness and the redness of snacks tended to increase [24] due to the Maillard reaction and the destruction of heat sensitive pigments. In this study, the SIP-F3 treatment and its protein fractions had the greatest differences in color; the use of an alkaline pH and high temperature possibly had the same effect on the destruction of sensitive pigments. In fact, during the adjustment of the pH to 11.0, the dispersion generated a sulfurous odor and a significant increase in viscosity.

### 3.5. Principal Component Analysis

**Figure 5:**
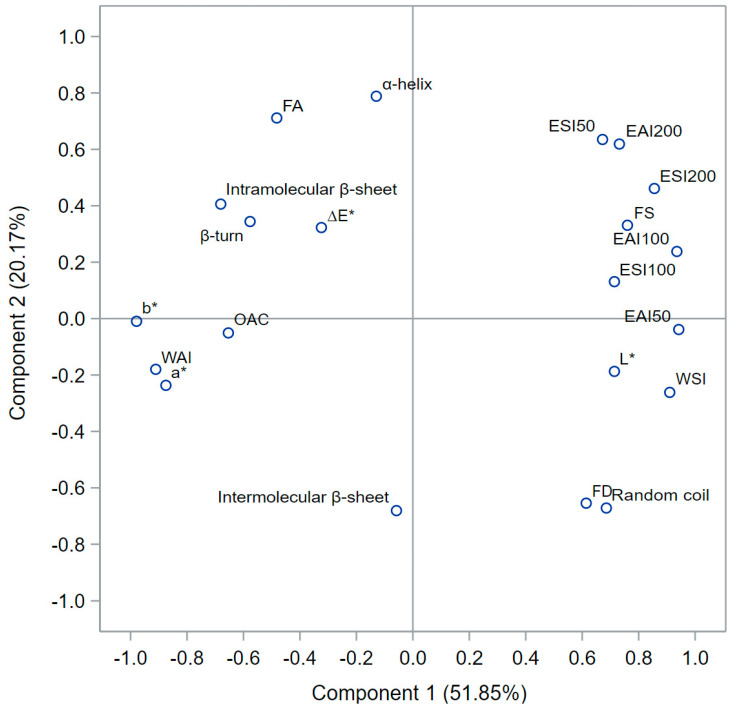
Principal component analysis (PCA) plot describing the relationship between the secondary protein structure and functional properties of Sacha Inchi protein fractions. OAC: oil absorption capacity; WAI: water absorption index; WSI: water solubility index; FA: foam activity; FS: foam stability; FD: foam density. L*, a*, b*, and ΔE: rectangular coordinates, and total difference of color parameters; EAI: emulsifying activity index and ESI: emulsifying stability index, at 50, 100, and 200 mg each.

A correlation-based principal component analysis (PCA) was performed to investigate the relationship between functional properties and the secondary structure of the SIPF, as shown in Table 6 and Figure 5.

The correlations were analyzed using a robust open-source software platform SAS^®^ OnDemand software(Cary, NC: SAS Institute Inc. Accessed in May 2023). The first two PC score plots explained 72.2% of the total variation. The first principal component (PC1) explained 51.85% of the total variation. The random coil, EAI50, EAI100, EAI200, ESI50, ESI100, ESI200, L*, FD, FS, and WSI, had large (>0.6) positive loadings, while the intramolecular β-sheet, β-turn, OAC, WAI, a*, b*, and FA had medium to large (−0.8 to −0.4) negative loadings, and the intermolecular β-sheet, α-helix, and ΔE had low (−0.2 to 0) negative loadings on PC1.

The correlation matrix values (Table 6) and PCA plot (Figure 5) provide insights into the relationship and strength of association between the functional properties and the secondary structure of proteins. In the correlation matrix (Table 6), high correlations were shaded to facilitate their analysis. The intermolecular β-sheet, intramolecular β-sheet, and β-turn structures exhibited a strong negative correlation with the EAI and ESI properties of the different SIPF. This could explain why the fractions with high percentages of these three structures (SIP-F5 and SIP-F7, Table 3) displayed low values of these properties, while those (SIP-F4 and SIP-F6, Table 3) that did not possess or have low percentages of these secondary structures, exhibited high values of these properties (Figure 4).

Furthermore, the β-turn and intramolecular β-sheet structures showed a strong positive correlation with the color attributes ΔE and WAI, while the random coil structures exhibited a high negative correlation with these same properties. This could explain why SIPF with high percentages of β-turn and intramolecular β-sheet structures and low levels of random coil structures, such as SIP-F3, SIP-F5, and SIP-F7 (Table 3), displayed significantly higher ΔE values (~7 to 14, Table 5) and higher WAI values (~3 to 5 *w*/*w*, Table 4) compared to the other fractions.

Similarly, the β-turn, β-sheet structures, and α-helix correlated positively with the FA attribute, which explains the high FA value of SIP-F1 (~132%, Table 4), compared to the other SIPF. On the other hand, the intramolecular β-sheet and β-turn structures had a strong positive correlation with the OAC and WAI properties, while the random coil structures had a high negative correlation with these functional properties. Thus, protein fractions with a high percentage of β-sheet and β-turn structures and low values of random coil structures, such as SIP-F3, SIP-F5, and SIPF-7 (Table 3), presented high OAC and WAI values (~7 to 9, and Table 4) compared to the other SIPF.

Lastly, the α-helix, β-turn, and intramolecular β-sheet structures had a strong negative correlation, while random coil structures had a strong positive correlation with the FD and WSI properties, as well as the L* color attribute. This explains why SIP-F4 and SIP-F6, which had low percentages of α-helix, β-turn, and intramolecular β-sheet structures and high percentages of random coil structures (Table 3), displayed significantly high values for FD (~63, Table 4), WSI (~87%, Table 4), and L* (~84, Table 5) compared to the other SIPF.

## 4. Conclusions

Sacha Inchi oil press-cake (*Plukenetia volubilis*) is a sustainable alternative for obtaining proteins. The experimental conditions evaluated in the present study were adequate to obtain various protein fractions from this by-product. Extraction with alkaline water (pH 11.0) at 65–70 °C was shown to be a promising method to obtain high extraction yields, related to the protein content and efficiency performance, and it can efficiently extract the majority of the proteins and peptides from SIPC. Additionally, the different protein fractions obtained promise to be suitable for different techno-functional properties of interest in the food and feed industries, as an alternative to the use of soybean meal. To our knowledge, this is the first report on the determination of the secondary structure content of SI protein fractions using the deconvolution/curve-fitting FTIR method, and its correlation analysis with the functional properties of these protein fractions. However, further studies focused, on the one hand, on the development of additional extraction steps to increase the purity of proteins with specific techno-functional properties, and on the other hand, on the evaluation of the digestibility and shelf life of extracts are needed to promote the valorization and use of Sacha Inchi protein, and consequently to contribute to the circular economy.

## Figures and Tables

**Table 1:** Proximal composition of Sacha Inchi oil press-cakes (wet basis).

Parameter	SIPC	SIPCD
Dry matter (%)	90.92 ^a^ ± 0.03	90.01 ^b^ ± 0.01
Protein (%)	54.47 ^b^ ± 0.11	56.86 ^a^ ± 0.42
Fat (%)	6.84 ^a^ ± 0.02	1.70 ^b^ ± 0.01
Total dietary fiber (%)	13.85 ^b^ ± 0.25	18.17 ^a^ ± 0.46
Ash (%)	6.08 ^b^ ± 0.02	6.38 ^a^ ± 0.02
Carbohydrates (%)	9.68 ^a^ ± 0.34	6.62 ^b^ ± 0.72

^a,b^ Different letters indicate significant differences among mean values of two columns (*p* < 0.05) (LSD Test). Sacha Inchi oil press-cake (SIPC) and defatted Sacha Inchi oil press-cake (SIPCD).

**Table 2:** Extraction efficiency of protein fractions from Sacha Inchi oil press-cake.

Treatment	Protein Content * (%, N × 5.71)	Extraction Yield (%)	Protein Recovery (%)	Protein Concentration Increase (Fold)
SIP-F1	60.53 ^d^ ± 0.00	4.89 ^e^ ± 0.36	5.21 ^e^ ± 0.38	1.06 ^d^ ± 0.00
SIP-F2	85.08 ^b^ ± 0.00	10.04 ^c^ ± 0.81	15.39 ^c^ ± 1.46	1.50 ^b^ ± 0.00
SIP-F3	83.94 ^b^ ± 0.00	24.73 ^a^ ± 0.06	36.50 ^a^ ± 0.09	1.48 ^b^ ± 0.00
SIP-F4	74.52 ^c^ ± 2.83	3.00 ^e^ ± 0.03	3.93 ^e^ ± 0.19	1.31 ^c^ ± 0.05
SIP-F6	74.23 ^c^ ± 0.00	2.17 ^e^ ± 0.12	2.83 ^e^ ± 0.16	1.31 ^c^ ± 0.00
SIP-F5	93.07 ^a^ ± 4.04	7.04 ^d^ ± 0.03	11.52 ^d^ ± 0.55	1.64 ^a^ ± 0.07
SIP-F7	83.37 ^b^ ± 0.00	22.56 ^b^ ± 0.12	33.08 ^b^ ± 0.18	1.47 ^b^ ± 0.00

^a–e^ Different letters indicate significant differences among values of the same column (*p* < 0.05) (LSD Test). SIP-F1 (0% NaCl; pH: 7.0; T 65–70 °C; 1 h extraction); SIP-F2 (5% NaCl; pH: 11.0; T 65–70 °C; 1 h extraction); SIP-F3 (0% NaCl; pH: 11.0; T 65–70 °C; 1 h extraction). SIP-F4 and SIP-F5: true albumins and true globulins of SIP-F2, respectively; SIP-F6 and SIP-F7: true albumins and true globulins of SIP-F3, respectively. * Wet basis.

**Table 3:** Sacha Inchi protein fractions’ amide I components.

Treatment	Intermolecular β-Sheet	Intramolecular β-Sheet	Random Coil	α-Helix	β-Turn
	1627–1610 cm^−1^	1642–1628 cm^−1^	1650–1643 cm^−1^	1659–1650 cm^−1^	1700–1660 cm^−1^
SIP-F1	3.95 ^c^ ± 3.30	28.71 ^bc^ ± 10.01	0.00 ^d^ ± 0.00	44.59 ^a^ ± 10.82	22.75 ^a^ ± 3.80
SIP-F2	19.53 ^a^ ± 2.49	0.00 ^e^ ± 0.00	57.69 ^b^ ± 6.22	0.00 ^d^ ± 0.00	22.78 ^a^ ± 6.78
SIP-F3	0.00 ^c^ ± 0.00	66.45 ^a^ ± 4.46	3.82 ^cd^ ± 6.61	0.42 ^d^ ± 0.74	29.31 ^a^ ± 1.97
SIP-F4	11.22 ^b^ ± 0.50	11.18 ^d^ ± 0.65	57.79 ^b^ ± 1.92	4.98 ^c^ ± 1.04	14.83 ^b^ ± 0.73
SIP-F6	11.95 ^b^ ± 1.10	0.00 ^e^ ± 0.00	70.61 ^a^ ± 2.31	0.00 ^d^ ± 0.00	17.43 ^b^ ± 1.20
SIP-F5	10.79 ^b^ ± 1.71	39.29 ^b^ ± 5.37	2.76 ^cd^ ± 3.88	18.94 ^b^ ± 7.31	28.22 ^a^ ± 10.51
SIP-F7	17.06 ^a^ ± 3.04	31.61 ^b^ ± 4.31	0.00 ^d^ ± 0.01	27.25 ^b^ ± 8.92	24.08 ^a^ ± 7.85

^a–e^ Different superscript letters indicate significant differences among values of different columns. (*p* < 0.05) SIP-F1 (0%NaCl; pH: 7.0; T 65–70 °C; 1 h); SIP-F2 (5% NaCl; pH: 11.0; T 65–70 °C; 1 h); SIP-F3 (0% NaCl; pH: 11.0; T 65–70 °C; 1 h); SIP-F4 and SIP-F5: true albumins and true globulins of SIP-F2, respectively; SIP-F6 and SIP-F7: true albumins and true globulins of SIP-F3, respectively.

**Table 4:** Techno-functional properties of Sacha Inchi protein fractions.

Treatment	OAC (*w*/*w*)	WAI (*w*/*w*)	WSI (%)	FA (%)	FS (%)	FD
SIP-F1	4.7 ^cd^ ± 0.6	0.7 ^d^ ± 0.0	14.9 ^b^ ± 0.0	133.4 ^a^ ± 3.5	85.8 ^a^ ± 0.9	18.1 ^c^ ± 2.5
SIP-F2	4.7 ^c^ ± 0.7	3.7 ^bc^ ± 0.4	15.8 ^b^ ± 1.3	87.9 ^d^ ± 4.7	65.6 ^b^ ± 5.0	35,6 ^b^ ± 3.4
SIP-F3	6.6 ^b^ ± 1.4	4.6 ^ab^ ± 0.6	4.7 ^c^ ± 1.2	97.7 ^c^ ± 2.6	61.8 ^b^ ± 5.6	27.1 ^bc^ ± 0.5
SIP-F4	4.3 ^d^ ± 0.4	0.5 ^d^ ± 0.1	87.5 ^a^ ± 1.4	36.4 ^f^ ± 0.0	89.4 ^a^ ± 6.2	63.6 ^a^ ± 0.0
SIP-F6	6.2 ^bc^ ± 0.4	0.7 ^d^ ± 0.1	86.3 ^a^ ± 1.0	56.8 ^e^ ± 2.6	86.2 ^a^ ± 1.5	61.6 ^a^ ± 1.1
SIP-F5	9.0 ^a^ ± 0.4	3.0 ^c^ ± 0.2	1.9 ^c^ ± 1.0	62.1 ^e^ ± 2.3	81.3 ^a^ ± 11.4	34.0 ^b^ ± 11.7
SIP-F7	7.9 ^ab^ ± 0.7	5.0 ^a^ ± 0.8	3.0 ^c^ ± 0.9	122.7 ^b^ ± 5.2	89.8 ^a^ ± 8.6	22.9 ^bc^ ± 0.8

^a–f^ Different superscript letters indicate significant differences among values of different columns (*p* < 0.05). SIP-F1 (0% NaCl; pH: 7.0; T 65–70 °C; 1 h); SIP-F2 (5% NaCl; pH: 11.0; T 65–70 °C; 1 h); SIP-F3 (0% NaCl; pH: 11.0; T 65–70 °C; 1 h); SIP-F4 and SIP-F5: true albumins and true globulins of SIP-F2, respectively; SIP-F6 and SIP-F7: true albumins and true globulins of SIP-F3, respectively. OAC: oil absorption capacity; WAI: water absorption index; WSI: water solubility index; FA: foam activity; FS: foam stability; FD: foam density.

**Table 5:** CIE-Lab color coordinates of Sacha Inchi protein fractions.

Treatment	L*	a*	b*	ΔE
SIPCD	85.55 ^a^ ± 0.13	−0.12 ^bc^ ± 0.00	12.04 ^b^ ± 0.01	0.0 ± 0.0
SIP-F1	84.62 ^a^ ± 0.10	−1.92 ^e^ ± 0.02	7.40 ^cd^ ± 0.01	5.06 ^bc^ ± 0.02
SIP-F2	84.45 ^a^ ± 0.01	−0.79 ^cd^ ± 0.00	10.55 ^bc^ ± 0.00	1.97 ^c^ ± 0.00
SIP-F3	77.94 ^b^ ± 3.15	−0.23 ^c^ ± 0.29	12.84 ^b^ ± 1.54	7.74 ^b^ ± 3.25
SIP-F4	84.12 ^a^ ± 1.46	−1.13 ^d^ ± 0.30	7.26 ^cd^ ± 0.97	5.30 ^bc^ ± 0.76
SIP-F6	83.89 ^a^ ± 1.42	−1.25 ^d^ ± 0.14	5.97 ^d^ ± 1.08	6.47 ^bc^ ± 1.40
SIP-F5	78.53 ^b^ ± 2.82	0.42 ^ab^ ± 0.22	11.75 ^b^ ± 2.19	7.42 ^b^ ± 2.52
SIP-F7	71.58 ^c^ ± 0.42	0.63 ^a^ ± 0.18	16.34 ^a^ ± 0.47	14.63 ^a^ ± 0.55

^a–e^ Different letters indicate significant differences among values of the same column (*p* < 0.05) (LSD Test). SIPCD: Sacha Inchi oil press-cake deffated; SIP-F1 (0% NaCl; pH: 7.0; T 65–70 °C; 1 h); SIP-F2 (5% NaCl; pH: 11.0; T 65–70 °C; 1 h); SIP-F3 (0% NaCl; pH: 11; T 65–70 °C; 1 h); SIP-F4 and SIP-F5: true albumins and true globulins of SIP-F2, respectively; SIP-F6 and SIP-F7: true albumins and true globulins of SIP-F3, respectively.

**Table 6:** Correlation matrix between functional properties and the secondary structure of Sacha Inchi protein fractions.

Functional Property	Protein Secondary Structures				
	Intermolecular β-Sheet	Intramolecular β-Sheet	Random Coil	A-Helix	β-Turn
OAC	0.027	0.498	−0.522	0.094	0.548
WAI	0.348	0.453	−0.460	0.003	0.262
WSI	0.008	−0.609	0.791	−0.426	−0.629
FA	−0.253	0.372	−0.707	0.714	0.370
FS	0.033	−0.407	0.252	0.246	−0.538
FD	0.284	−0.509	0.769	−0.643	−0.451
L*	−0.022	−0.624	0.588	−0.109	−0.280
a*	0.219	0.598	−0.515	−0.070	0.426
b*	0.113	0.689	−0.694	0.137	0.500
ΔE	0.054	0.459	−0.404	0.140	0.029
EAI50	−0.048	−0.583	0.667	−0.229	−0.543
EAI100	−0.156	−0.516	0.459	0.068	−0.508
EAI200	−0.315	−0.337	0.083	0.469	−0.280
ESI50	−0.588	−0.113	0.021	0.369	−0.142
ESI100	−0.201	−0.403	0.456	−0.124	−0.256
ESI200	−0.411	−0.345	0.235	0.254	−0.250

The interpretation of correlation values in the context of the interactions between functional property and protein secondary structure follows the following guide: a correlation value of r = ±1 indicates a high positive/negative correlation. A correlation value of r = ±0.25–0.5 indicates a moderate positive/negative correlation. A correlation value of r = ±0–0.25 indicates a low positive/negative correlation. OAC: oil absorption capacity; WAI: water absorption index; WSI: water solubility index; FA: foam activity; FS: foam stability; FD: foam density. L*, a*, b*, and ΔE: rectangular coordinates, and total difference of color parameters; EAI: emulsifying activity index and ESI: emulsifying stability index, at 50, 100, and 200 mg each.

## Data Availability

The data are not publicly available due to restrictions of the project; they are included in the paper.
